# A rarefaction approach for measuring population differences in rare and common variation

**DOI:** 10.1093/genetics/iyad070

**Published:** 2023-04-19

**Authors:** Daniel J Cotter, Elyssa F Hofgard, John Novembre, Zachary A Szpiech, Noah A Rosenberg

**Affiliations:** Department of Genetics, Stanford University, Stanford, CA 94305, USA; Institute for Computational and Mathematical Engineering, Stanford University, Stanford, CA 94305, USA; Department of Human Genetics, University of Chicago, Chicago, IL 60637, USA; Department of Biology, Pennsylvania State University, University Park, PA 16802, USA; Institute for Computational and Data Sciences, Pennsylvania State University, University Park, PA 16802, USA; Department of Biology, Stanford University, Stanford, CA 94305, USA

**Keywords:** common variants, rare variants, rarefaction, sample-size correction

## Abstract

In studying allele-frequency variation across populations, it is often convenient to classify an allelic type as “rare,” with nonzero frequency less than or equal to a specified threshold, “common,” with a frequency above the threshold, or entirely unobserved in a population. When sample sizes differ across populations, however, especially if the threshold separating “rare” and “common” corresponds to a small number of observed copies of an allelic type, discreteness effects can lead a sample from one population to possess substantially more rare allelic types than a sample from another population, even if the two populations have extremely similar underlying allele-frequency distributions across loci. We introduce a rarefaction-based sample-size correction for use in comparing rare and common variation across multiple populations whose sample sizes potentially differ. We use our approach to examine rare and common variation in worldwide human populations, finding that the sample-size correction introduces subtle differences relative to analyses that use the full available sample sizes. We introduce several ways in which the rarefaction approach can be applied: we explore the dependence of allele classifications on subsample sizes, we permit more than two classes of allelic types of nonzero frequency, and we analyze rare and common variation in sliding windows along the genome. The results can assist in clarifying similarities and differences in allele-frequency patterns across populations.

## Introduction

The study of data on genetic variation often begins with simple questions. Which alleles are present? In which populations are they present, and where are they absent? Which alleles are common, and which are rare? Often, the first calculations that an analyst performs on a population-genetic dataset seek to answer such questions.

To take one example, a recent study of Witt *et al.* (2022) sought to characterize genetic variation in modern and archaic populations, with a particular interest in the sharing of alleles among groups. In their Fig. 5, Witt *et al.* (2022) tabulated, for alleles classified as archaic, the fractions of those alleles that appear in modern Europeans, South Asians, and East Asians, in pairs among these three groups, and in all three groups.

In studies of the presence and absence of alleles in populations, differing sample sizes among the groups can influence the resulting assessments. For example, an allele absent in a small sample might eventually be found in a larger sample, so that a population with a sample size that is small might appear to possess fewer alleles than a population with one that is large. This problem is addressed by the rarefaction method, borrowed for population genetics (e.g. Kalinowski 2004) from ecological work on species diversity (Hurlbert 1971; Gotelli and Colwell 2001). Using a combinatorial formula, given sample size $N_{j}$ for population *j* and a fixed value of $g\leq N_{j}$, all possible subsamples of size *g* are considered, and the expected number of distinct alleles across random samples of size *g* is calculated. Multiple populations of different sample size can be compared by examining subsamples of equal size *g*.

Kalinowski (2004) devised a rarefaction-based calculation of “private allelic richness,” a measure of the fraction of alleles that are private to a particular population—considering subsamples of size *g* from each of a series of populations. Generalizing this concept, Szpiech *et al.* (2008) introduced a calculation of the fraction of alleles that are private to a *set* of populations—that is, found in each of the populations—when subsamples of size *g* are taken in each population. Szpiech *et al.* (2008) examined geographic distributions of alleles in samples from multiple populations, all standardized with the same subsample size *g*. Thus, for example, for Populations 1, 2, and 3, with different sample sizes, the rarefaction-based calculation enables a comparison of the fraction of alleles found only in 1, only in 2, only in 3, in 1 and 2 but not 3, in 1 and 3 but not 2, in 2 and 3 but not 1, and in all three groups—assuming that all three groups have subsamples of equal size.

Recently, Biddanda *et al.* (2020) introduced a new computation and visualization to compare the presence and absence of alleles across populations. Seeking to describe geographic distributions of alleles across multiple populations—as in Szpiech *et al.* (2008) and Witt *et al.* (2022)—Biddanda *et al.* (2020) made an additional distinction between alleles that are present and *rare* and those that are present and *common*. For each of several populations, they classified alleles into three categories: rare, common, and unobserved. For a population set, they tabulated fractions of alleles that possess particular classes, illustrating the classifications in new visualizations.

In the same way that sample size can affect presence and absence, sample size can also affect the classification of an allele as present and rare as opposed to present and common. Suppose a locus has the same allele frequencies in Populations 1 and 2, with sample sizes 39 and 40, respectively. Suppose a maximum of 5% is the largest allele frequency classified as rare. An allele *A* of frequency 5% that is regarded as rare in an infinite population will be regarded as rare in Population 1 when 1 copy is observed in the sample of size 39. The probability of observing exactly 1 copy is $\left(\begin{array}{c} 39\\ 1 \end{array}\right)\left(0.05^{1}\right)\left(0.95^{38}\right)\approx 0.278$. The allele will be regarded as rare in Population 2 if 1 copy is observed *or* if 2 copies are observed. The associated probability is $\left(\begin{array}{c} 40\\ 1 \end{array}\right)\left(0.05^{1}\right)\left(0.95^{39}\right)+\left(\begin{array}{c} 40\\ 2 \end{array}\right)\left(0.05^{2}\right)\left(0.95^{38}\right)\approx 0.548$. Hence, as a result of different sample sizes, the two populations have the potential to differ dramatically in the number of their truly rare alleles (that is, rare at the population level) that are classified as rare in samples.

Here, we extend the geographic classification of alleles into categories of rare, common, and unobserved, as in Biddanda *et al.* (2020), but accounting for differences in sample size, as in Szpiech *et al.* (2008). In particular, we extend the rarefaction approach from Szpiech *et al.* (2008), which only considered presence and absence, to account for the three categories of Biddanda *et al.* (2020): unobserved, rare, and common. We examine whether the rarefaction correction to make use of equal sample sizes in the data of Biddanda *et al.* (2020) influences the interpretation of rare and common human variation. In the spirit of Biddanda *et al.* (2020), we also include a variety of visualizations for understanding sample-size-corrected patterns in the geographic distributions of rare and common alleles.

## Statistical methods

Consider a single locus in an individual. We henceforth use “allelic type” to refer to one of a set of possible variants at a locus and “allele” to refer to an observation at a given locus in a single individual. Considering a locus with I≥2 allelic types, we denote by $N_{ij}$ the number of copies of allelic type *i* observed in a sample from population *j*. By extension, $N_{j}=\sum_{i=1}^{I}N_{ij}$ is the sample size of population *j* at the locus. We consider J≥2 populations.

Biddanda *et al.* (2020) declare “rare” allelic types as those with nonzero frequency less than or equal to 100z% in a population, where *z* is a specified numerical cutoff (they use z=0.05). They then classify allelic types with frequency greater than 100z% as “common.” This classification gives rise to their three frequency categories of unobserved, rare, and common. Thus, considering all *J* populations, an allelic type takes on a “pattern” denoted by $x=(x_{1},x_{2},\ldots ,x_{J})$, where each $x_{j}$ has a value in {unobserved, rare, common}, herein shortened to {U,R,C}.

### Three allelic classes: unobserved, rare, and common

For a sample with counts $N_{ij}$ for the *I* allelic types in the *J* populations, we consider subsamples with specified sizes. Suppose that a sample of size *g* alleles is drawn in each of the *J* populations, for a total sample size of *Jg*. We calculate the probability that when we draw a sample of size *Jg*, an allelic type has pattern x.

The probability $U_{ijg}$ that allelic type *i* is *unobserved* in a subsample of size *g* from population *j* is


(1) Uijg=(Nj−Nijg)(Njg).

Here, the numerator is the number of ways to draw *g* alleles from among the alleles that *do not* have allelic type *i*. The denominator is the total number of ways to draw *g* alleles from among the $N_{j}$ alleles in population *j*.

The probability $R_{ijg}$ that allelic type *i* is *rare* in a subsample of size *g* is the probability of observing at least 1 and at most $\left\lfloor zN_{j}\right\rfloor$ copies of allelic type *i* in a subsample of size *g*. The floor function accounts for the classification of an allelic type with frequency exactly 100z% as rare rather than common. The probability $R_{ijg}$ satisfies


(2) Rijg=∑k=1⌊zNj⌋[(Nijk)(Nj−Nijg−k)](Njg).

The numerator in equation 2 sums over all possible ways to choose at least 1 and at most $\left\lfloor zN_{j}\right\rfloor$ copies of allelic type *i*. The denominator again gives the total number of ways to draw *g* alleles from the population sample size $N_{j}$.

Finally, the probability that allelic type *i* is *common* in a sample of size *g* taken from population *j* is simply


(3) $$C_{ijg}=1-U_{ijg}-R_{ijg}.$$

Now that we have probabilities for an allelic type in a single population, we consider all *J* populations to determine the probability of a particular pattern x. The probability that allelic type *i* has pattern $x=(x_{1},x_{2},\ldots ,x_{J})$ in a sample containing *g* alleles each from the *J* populations is


(4) $$\prod_{\;j=1}^{J}f_{ijg}(x_{j}),\;\text{where}\;f_{ijg}(x_{j})=\left\{ \begin{array}{cc} U_{ijg}, & x_{j}=\text{U},\\ R_{ijg}, & x_{j}=\text{R},\\ C_{ijg}, & x_{j}=\text{C}. \end{array}\right.$$

At a locus, we sum across all *I* allelic types to give the expected fraction of allelic types that have pattern x:


(5) $$\frac{1}{I}\sum_{i=1}^{I}\prod_{\;j=1}^{J}f_{ijg}(x_{j}).$$

### Extension to more than three classes

We can generalize the results describing unobserved, rare, and common allelic types to compute the probability $P_{ijg}$ of finding an allelic type *i* in population *j* in a specified frequency window, where arbitrarily many windows are permitted. Define a window $\left(z_{1},z_{2}\right]$, describing allelic types with a frequency greater than $z_{1}$ and less than or equal to $z_{2}$. Equation 2 for the probability that a sample of size *g* has its frequency for allelic type *i* in the window (0,z] generalizes, and the probability that allelic type *i* has its frequency in $\left(z_{1},z_{2}\right]$ is


(6) Pijg=∑k=⌊z1Nj⌋+1⌊z2Nj⌋[(Nijk)(Nj−Nijg−k)](Njg).

Equation 6 can consider arbitrary divisions of the unit interval for frequencies into disjoint intervals. Note that if we instead regard intervals as having a closed lower bound and an open upper bound, so that we consider the probability that an allelic type has frequency in $\left[z_{1},z_{2}\right)$, then we simply change the limits of the sum to $\left\lceil z_{1}N_{j}\right\rceil$ and $\lceil z_{2}N_{j}\rceil -1$.

### Biallelic loci

For biallelic loci, I=2, suppose we are interested in only one specific allelic type. We label this allelic type by 1 and the other allelic type by 2 and write simplified formulas for $U_{ijg}$ and $R_{ijg}$. $N_{1j}$ is the count of allelic type 1 in population *j* and $N_{2j}$ is the count of allelic type 2. Then


(7) Uijg=(N2jg)(Njg),

(8) Rijg=∑k=1⌊zNj⌋[(N1jk)(N2jg−k)](Njg).

Equation 4 can then be used to calculate the probability that allelic type 1 has pattern $x=(x_{1},x_{2},\ldots ,x_{J})$. With three frequency classes in each of *J* populations, allelic type 1 has $3^{J}$ possible patterns.

## Data analysis

### Biddanda *et al.* (2020) dataset

Biddanda *et al.* (2020) used data from the 2,504 individuals in the 26 populations of the 1000 Genomes Project (The 1000 Genomes Project Consortium 2015; Byrska-Bishop *et al.* 2022) to explore the relative abundances of different patterns x, considering five “super-populations.” They used the globally minor allele at each locus—the allelic type at global frequency less than 50%—to classify each locus as a pattern $x=(x_{1},x_{2},\ldots ,x_{J})$, where J=5.

They placed the 1000 Genomes populations, annotated here by three-letter abbreviations, into the five super-populations. From 1 to 5, vector entries correspond to African (ESN, GWD, LWK, MSL, YRI), European (FIN, GBR, IBS, TSI), South Asian (BEB, GIH, ITU, PJL, STU), East Asian (CDX, CHB, CHS, JPT, KHV), and American super-populations (ACB, ASW, CEU, CLM, MXL, PEL, PUR). Thus, for example, a locus rare in Africa, common in East Asia, and unobserved elsewhere has pattern x={R, U, U, C, U} or RUUCU for short.

Biddanda *et al.* (2020) considered genome-wide biallelic SNPs, classifying each SNP into one of $3^{5}-1$ patterns based on the globally minor allele; because each locus is polymorphic by definition, the pattern UUUUU is omitted in their analysis.

We downloaded the dataset used by Biddanda *et al.* (2020) from the 1000 Genomes FTP server (see “Data availability”). We retained the same super-population categories used by Biddanda *et al.* (2020). After filtering to consider only biallelic SNPs, we determined the globally minor allele for each SNP. Our definition of the minor allele is the allelic type that, when averaging relative frequencies across the five super-populations, has frequency below $\frac{1}{2}$; for 240 sites genome-wide with exactly 50% global frequency for each of the two allelic types, we chose one allelic type at random to be the “minor” allele. We then tabulated counts of the minor allele for the five super-populations, disregarding sites for which data were entirely missing in at least one of the five. This process left us with 95,563,258 SNPs in the 2,504 individuals.

### Pointwise rarefaction analysis

To evaluate the effect of sample-size correction on the geographic distribution of allelic types, we applied the rarefaction calculation (equation 4) to the 1000 Genomes SNPs in the five super-populations. This calculation relies on the biallelic equations 7 and 8, along with equation 3. For an illustrative analysis, we considered 1,226,225 SNPs on chromosome 22, ensuring that each SNP possessed a sample of size 500 or greater in each of the five super-populations (the equivalent of 250 diploid individuals).

Thus, for each of a series of values of *g*, for each SNP, focusing on the minor allele, we obtained probabilities for each of the $3^{5}=243$ patterns, treating 5% as the maximal frequency for allelic types treated as rare. For fixed *g*, for each of the 243 patterns, we averaged the SNP-specific probabilities across all SNPs to determine the mean probability that a randomly chosen locus in the SNP set has a specific pattern. To understand the effect of the subsample size on pattern probabilities, we modulated the sample size *g* in increments of 10, considering all multiples of 10 in [10,500].

Next, to study the numbers of super-populations in which variants are common and rare, we collapsed the 243 patterns into summaries that disregard the identities of the super-populations in which allelic types are unobserved, rare, and common. For these summaries, we track only the numbers of U’s, R’s, and C’s for a given allelic type as an ordered triple (|U|,|R|,|C|). For example, if an allelic type has the pattern RUUUU, URUUU, UURUU, UUURU, or UUUUR, then it is summarized as (4,1,0). The number of possible summaries is 21.

### Sliding-window analysis

To examine the change in pattern probabilities along the genome, we calculated the probability distribution of patterns in sliding windows. We tiled the genome with nonoverlapping 100-kb windows. Within each window, we averaged the 21 summaries across SNPs within the window, still focusing on the globally minor allele at each SNP. For this analysis, we focused on a single value of *g*, choosing g=500, summarizing the patterns using the 21 ordered triples (|U|,|R|,|C|).

## Results

### Pointwise rarefaction analysis

**Fig. 1.**
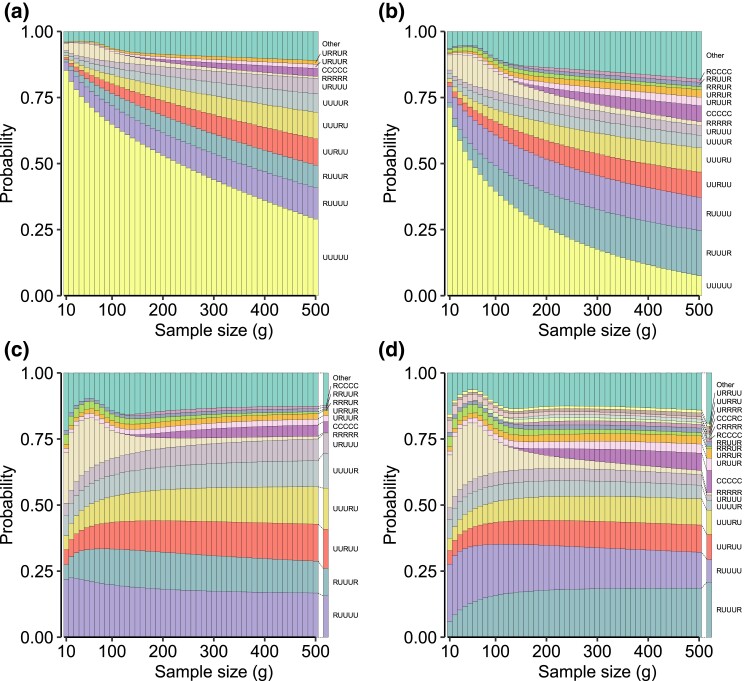
Probability that the globally minor allele at a locus has a given geographic distribution pattern as a function of *g*, the number of alleles sampled in each super-population (equation 4). a) All SNPs on chromosome 22. b) All nonsingleton SNPs on chromosome 22. c) All SNPs on chromosome 22, normalizing by 1−P[UUUUU]. d) All nonsingleton SNPs on chromosome 22, normalizing by 1−P[UUUUU]. In a five-letter pattern, U is unobserved, R is rare (>0% and ≤5% population frequency), and C is common (>5%). The order in which super-populations are listed is Africa, Europe, South Asia, East Asia, and the Americas. For example, RUUUU refers to a minor allele that is rare in Africa and unobserved in each of the other four super-populations.

Figure 1a shows the pointwise probabilities of the various patterns for 1,226,225 SNPs on chromosome 22. The figure visualizes the 11 patterns that have probability 1% or greater at g=250, grouping the other 232 patterns into a single “other” category; this choice of the intermediate value of g=250 facilitates visualization of patterns that are probable at high *g* or low *g* but not both.

The highest-frequency pattern for all sample sizes is UUUUU, the probability of observing no variation across the five super-populations; this pattern is the one most likely to be observed if an allelic type is present in the full data but extremely rare. Among the other high-frequency patterns, five of the next six represent allelic types that are rare in one super-population and absent in the other four; the sixth, RUUUR, is allelic types rare in both the African and American super-populations, likely a result of admixed African-descended populations in the American super-population. CCCCC is included; it is the only high-frequency pattern that includes any common variation.

Increases in the sample size decrease the frequency of UUUUU and increase the frequencies of patterns containing rare allelic types. As the sample size increases, the probability increases that a rare variant is detected in a sample, so that previously unobserved allelic types are increasingly likely to be observed as rare.

In Fig. 1b, we analyze the effect of extremely rare allelic types on the patterns by discarding all 614,354 SNPs whose minor allele appears only once among the 2,504 individuals, leaving 611,871 nonsingleton SNPs on chromosome 22. By removing singletons, we deflate the UUUUU proportion, revealing patterns that previously grouped into the “other” category; the number of patterns with frequency at least 1% at g=250 increases from 11 to 14. All 11 previous high-frequency patterns are observed, in addition to two in which allelic types are rare in multiple super-populations and unobserved in others (RRRUR, RRUUR) and one in which allelic types are common in some super-populations and rare in others (RCCCC). Patterns containing one R and four U’s continue to be among the higher-frequency patterns, indicating that these patterns result from rare variation that is not limited to allelic types present in only a single copy. Similar observations hold genome-wide (Supplementary Fig. S1).

In Biddanda *et al.* (2020), without a sample-size correction, all loci are biallelic. Hence, no variant can be entirely unobserved, and Biddanda *et al.* (2020) did not consider the UUUUU pattern. To facilitate a comparison of the relative probabilities of the remaining 242 patterns between our analysis and that of Biddanda *et al.* (2020), we remove the UUUUU pattern at each *g* and divide the remaining pattern frequencies by 1−P[UUUUU] (Fig. 1c). With this normalization, three patterns with frequencies below 1% at g=250 in Fig. 1a now have frequencies greater than or equal to 1%: RRUUR, RCCCC, and RRRUR. For most patterns, the frequency is largely unaffected by changes in the sample size *g*. An interesting exception is RRRRR, for which a particularly strong effect of the discrete sample size is evident. At small *g*, this pattern is observed when exactly one copy of an allelic type is seen in each of the five super-populations; as described in the example in the Introduction, common allelic types with frequencies near the frequency cutoff between rare and common are mistakenly categorized as rare, and as the sample size *g* increases, it is possible to correctly determine that those allelic types are, in fact, common.

Figure 1c provides a comparison of the sample-size-corrected probabilities with the empirical pattern frequencies observed in the sample, the frequencies that correspond to the non-sample-size-corrected calculation of Biddanda *et al.* (2020) (rightmost column of Fig. 1c). Although many of the corrected pattern frequencies differ from the uncorrected frequencies at small *g*, at g=500, the sample-size-corrected probability of observing a given pattern is comparable to the empirical frequency of that pattern in the full set of loci. A similar general agreement of the empirical frequency to sample-size-corrected probabilities at high *g* is observed in Fig. 1d, with singletons excluded. As in the comparison of Fig. 1b and a, exclusion of singletons increases the number of patterns occurring at frequency ≥0.01 when g=250, from 13 to 18. The exclusion of singletons reduces frequencies for patterns with one R and four U’s, so that additional patterns cross the 1% threshold.

**Fig. 2.**
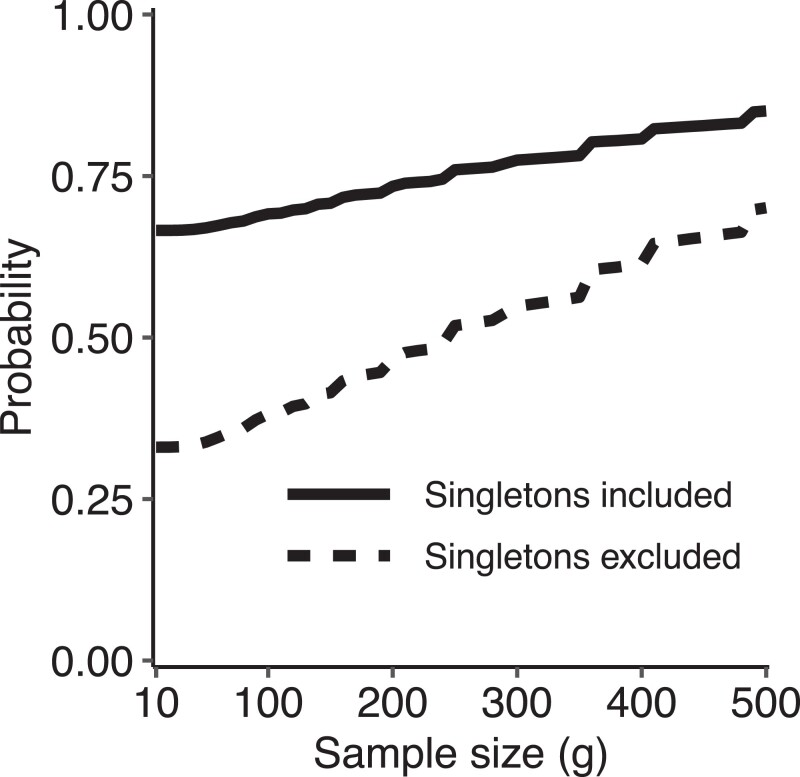
Probability as a function of the sample size *g* that across SNPs on chromosome 22, the highest-probability non-UUUUU pattern calculated using a sample-size correction (equation 4) matches the empirically observed pattern without sample-size correction.

A further comparison of sample-size-corrected pattern frequencies with uncorrected frequencies appears in Fig. 2. In this figure, we evaluate the fraction of loci for which the empirical pattern at a locus matches the (non-UUUUU) pattern with greatest sample-size-corrected probability. Performing this computation at each value of the sample size *g*, we observe that the probability that the empirical pattern is a match to the highest-probability pattern with sample-size correction increases with *g* (Fig. 2). With singletons included, at g=10, the probability of agreement is 66.6%, and at g=500, it is 85.1%. The probabilities are somewhat lower with singletons excluded; at g=10, the agreement probability is 33.0%, and at g=500, it is 70.1%. Because singleton loci can only take on one non-UUUUU pattern in the rarefaction calculation (rare in the one super-population where the allelic type is seen), given that they must be polymorphic in the empirical data, the empirical pattern necessarily agrees with the highest-probability sample-size-corrected non-UUUUU pattern.

**Fig. 3.**
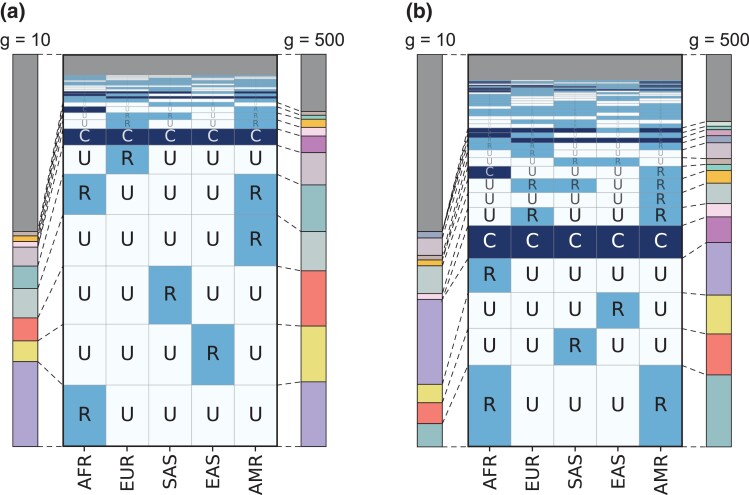
Pattern probabilities at g=10 and g=500 compared to non-sample-size-corrected pattern probabilities. The sample-size-corrected and non-sample-size-corrected probabilities are calculated on chromosome 22. a) All SNPs on chromosome 22, as in Fig. 1c, with non-sample-size-corrected pattern probabilities depicted analogously to Fig. 3b of Biddanda *et al.* (2020). b) Nonsingleton SNPs on chromosome 22, as in Fig. 1d, with non-sample-size-corrected pattern probabilities depicted analogously to Fig. 3c of Biddanda *et al.* (2020). The colors used to depict pattern probabilities for g=10 and g=500 are the same as those used in Fig. 1.

We accentuate the comparison between sample-size-corrected and uncorrected pattern frequencies by depicting the non-UUUUU pattern frequencies at g=10 and g=500, alongside depictions of corresponding empirical pattern frequencies in the style of Biddanda *et al.* (2020) (Fig. 3). At the smaller g=10, common variation is unlikely: allelic types at the low end of the frequency interval for common variation are relatively unlikely to be sampled in such a small sample size, so that pattern CCCCC has a low probability. However, at g=500, allelic types that are truly common are more likely to be detected as common. The pattern frequencies for large *g* generally agree with the empirical pattern frequencies without sample-size correction.

**Fig. 4.**
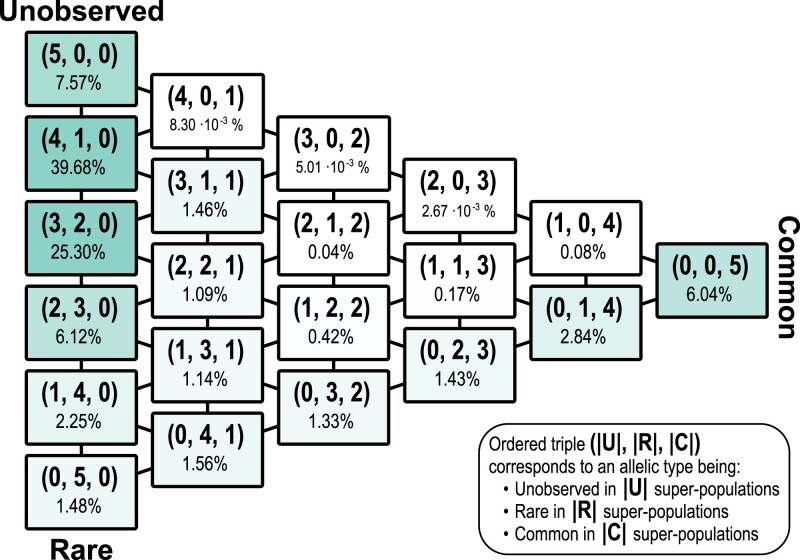
Probabilities for groups of patterns for a nonsingleton minor allele on chromosome 22, in samples containing g=500 alleles from each super-population. The figure summarizes the g=500 column of Fig. 1b, tabulating the numbers of super-populations in which allelic types are unobserved, rare, and common. An ordered triple is written (|U|,|R|,|C|), so that, for example, 2.84% for the entry (0,1,4) indicates that 2.84% of allelic types are unobserved in 0 super-populations, rare in 1 super-population, and common in 4 super-populations.

Figure 4 provides a summary of pattern frequencies at g=500, collapsing the 243 patterns into 21 groups tabulating the numbers of super-populations in which allelic types are unobserved, rare, and common. Considering all 243 patterns and excluding singletons as in Fig. 1b, we observe, as can be seen in Fig. 1b, that the highest probabilities occur for groups (4,1,0), (3,2,0), (5,0,0), and (2,3,0), representing allelic types that are rare or unobserved in all super-populations. Next in probability is (0,0,5), representing allelic types that are common in all super-populations. Probabilities are particularly small for scenarios (4,0,1), (3,0,2), (2,0,3), and (1,0,4), representing variation that is common in some super-populations and unobserved in others.

### Sliding-window analysis

**Fig. 5.**
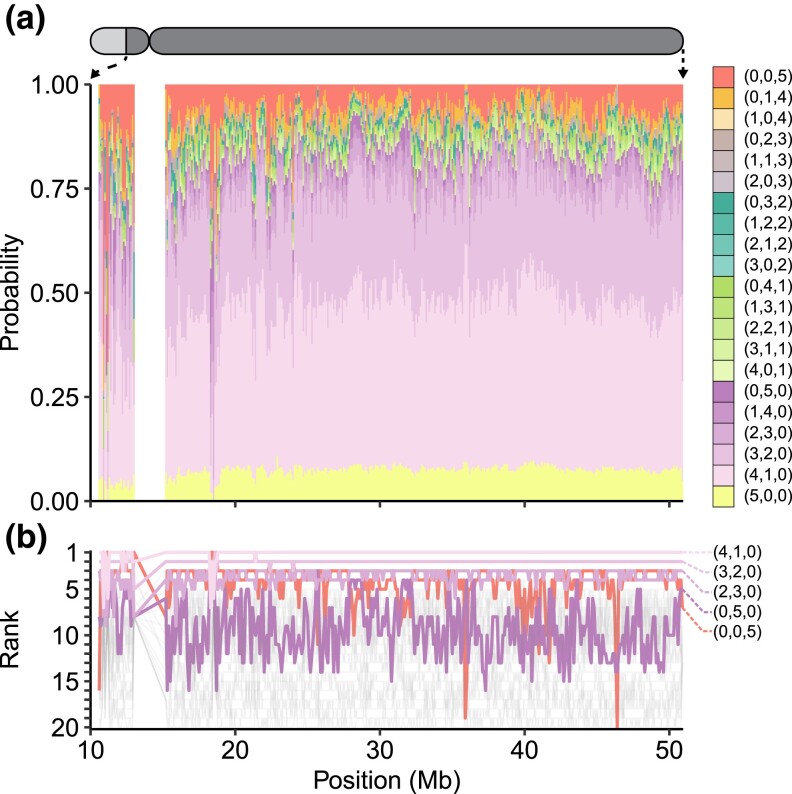
Probabilities for groups of patterns for minor alleles on chromosome 22, in samples containing g=500 alleles from each super-population, averaged across all nonsingleton loci in nonoverlapping 100-kb sliding windows. Ordered triples are written (|U|,|R|,|C|), with the entries representing the numbers of super-populations in which allelic types are unobserved, rare, and common, respectively. Triples are grouped by color, varying within classes with a given number of super-populations in which allelic types are common. a) Probabilities for pattern groups. b) Local frequency ranks of pattern groups, from 1 to 20 (the pattern in which allelic types are unobserved in all super-populations, (5,0,0), is excluded). For simplicity, only those pattern groups that achieve frequency rank 1 or 2 in at least one window on the chromosome receive a color. The remaining pattern groups are shaded gray. Note that the first 10 Mb of chromosome 22 are excluded, as they do not appear in the 1000 Genomes dataset; the centromere is also excluded.

Figure 5a shows the 21 groups of patterns as a function of genomic position in 100-kb, nonoverlapping windows on chromosome 22, considering nonsingleton loci and samples of size g=500. In general, the probability distribution of the 21 groups shows little variation across the chromosome, mimicking the pointwise observations in Fig. 4. The highest-probability pattern groups are generally those that represent allelic types that are rare in one or more super-populations and unobserved in the others. A relatively high probability also occurs for allelic types that are common in all five super-populations.

Figure 5b visualizes changes in rank for the groups of patterns as a function of position along the chromosome, highlighting the pattern groups that enter the top two ranks in at least one window. This visualization emphasizes that patterns in which allelic types are rare in one or two super-populations have the highest frequency in most windows. It also uncovers windows that show a difference from the chromosome-wide average. For example, between 18 and 19 Mb, a spike occurs in the probability that a minor allele is common in all five super-populations, and the group (0,0,5), which often lies at rank 3, instead jumps to rank 1.

**Fig. 6.**
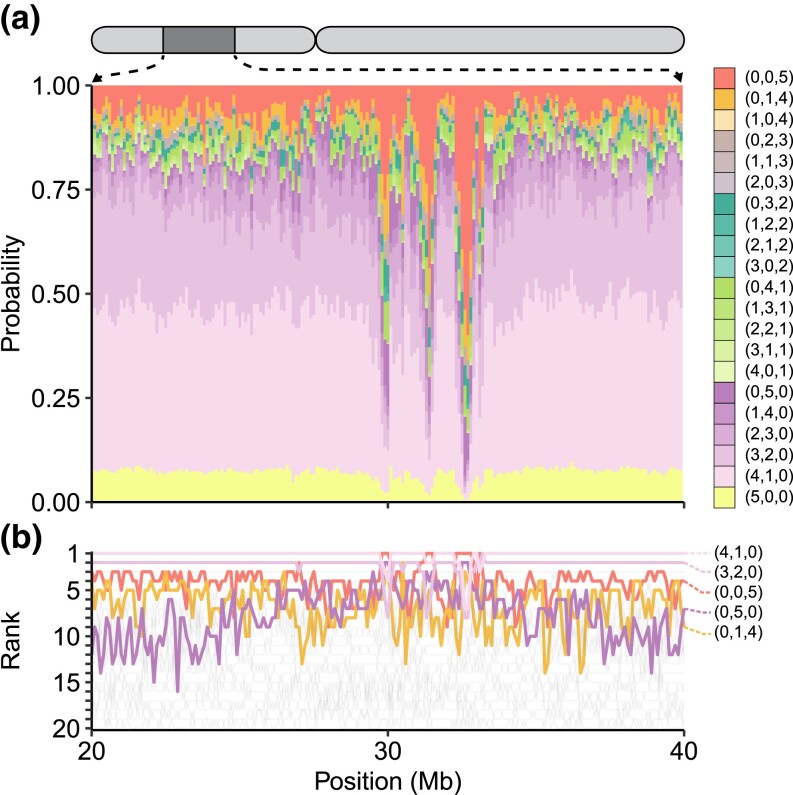
Probabilities for pattern groups for minor alleles of nonsingleton loci appearing between 20 and 40 Mb on chromosome 6, covering the HLA region (approximately 28.5–33.5 Mb on reference build hg38). The data analysis and figure design follow Fig. 5. a) Probabilities for pattern groups. b) Local frequency ranks of pattern groups.

To illustrate one of many deviations from typical pattern probabilities that occur periodically across the genome (Supplementary Fig. S2), we consider an example. In particular, as local changes in the extent to which allelic types are globally common can reflect evolutionary processes such as balancing selection, we examine the local change in probabilities in the highly variable HLA region on chromosome 6 (Fig. 6), where balancing selection is an important phenomenon (Meyer *et al.* 2018). Interestingly, in the HLA region (28.5–33.5 Mb), the group (0,0,5) has rank 1 in many windows, as might be expected for a region in which a balancing selection process maintains nontrivial frequencies for allelic types across many populations.

## Discussion

We have introduced a method for obtaining sample-size-corrected pattern probabilities describing the geographic distribution of allelic types. The method combines the “Geovar” plots of Biddanda *et al.* (2020)—which describe the probabilities with which allelic types are unobserved, rare, or common in different population groups—with the rarefaction approach of Szpiech *et al.* (2008), which mathematically studies geographic distributions of allelic types in subsamples that have an equal size in different groups.

Our analysis finds that with the use of a parameter *g* for the fixed sample size examined in each of the various groups, probabilities of allelic patterns do change somewhat (Fig. 1). Most notably, as *g* increases, the probability of classifying an allelic type as entirely unobserved declines (Fig. 1a and b). With this pattern omitted, pattern probabilities are relatively stable with *g* (Fig. 1c and d). However, *g* must be sufficiently large before the stability emerges. In small samples, discreteness effects influence the probability that an allelic type is rare in all groups; in using the rarefaction approach to examine pattern probabilities, such effects can potentially be mitigated by increasing the maximal frequency regarded as rare in small samples. Such an approach might be warranted in cases in which some of the groups of interest have samples that are much smaller than those of other groups, such as in comparisons involving ancient and modern data; suitable choices of frequency thresholds will depend on the specific sample sizes in data sets and on the underlying distribution of true allele frequencies. Conversely, if all sample sizes are extremely large, it may be convenient to use equation 6 to distinguish multiple tiers of rare allelic types, for example, for separating frequency classes rare enough to be restricted to one group from higher-frequency classes whose allelic types are still rare but likely to be found in multiple populations.

With a large sample size, g=500, our pattern probabilities with a sample-size correction closely match those observed without a sample-size correction in the manner of Biddanda *et al.* (2020) (Fig. 3). This general agreement suggests that the sample sizes in the Biddanda *et al.* (2020) super-population assignment—504, 404, 489, 504, and 603 individuals for AFR, EUR, SAS, EAS, and AMR, respectively—are sufficiently large that differences among them likely had little effect on the non-sample-size-corrected pattern probabilities of Biddanda *et al.* (2020) when using 5% as the demarcation between rare and common allelic types. In particular, our pattern probability calculations with sample-size corrections recapitulate the finding that most allelic types are rare in one or a few super-populations and unobserved in the others, or common in all super-populations (Fig. 4).

The work of Biddanda *et al.* (2020) is motivated by a goal not only of describing features of human genetic similarity and difference, it is also one of many examples of studies that place particular emphasis on new visualizations to capture those features (e.g. Mountain and Ramakrishnan 2005; Conrad *et al.* 2006; Pickrell *et al.* 2009; Teo and Small 2010; Rosenberg 2011; San Lucas *et al.* 2012; Petkova *et al.* 2016; Marcus and Novembre 2017; Diaz-Papkovich *et al.* 2019; Greenbaum *et al.* 2019; Peter *et al.* 2020; Battey *et al.* 2021). Such visualizations provide new representations of population-genetic statistics for use in understanding processes that affect genetic variation across populations. Emphases on visualization have been of increasing interest in light of ongoing misrepresentations of human population-genetic findings—particularly the misuse of graphical visualizations as apparent evidence of unsupportable views of human difference belied by the analyses that underlie the graphics (Carlson *et al.* 2022). Pattern probabilities, such as those we have considered here and those of Biddanda *et al.* (2020), enable a variety of visualizations of human variation beyond the “Geovar” style. Our Fig. 1, describing pattern probabilities in the categories “unobserved,” “rare,” and “common,” updates visualizations of the sample-size-corrected pattern probabilities of Szpiech *et al.* (2008), which grouped rare and common allelic types in a single category of “observed” allelic types. Figure 4, summarizing pattern probabilities by the number of super-populations in which allelic types are unobserved, rare, and common, updates similar summaries that also did not distinguish between rare and common allelic types (Rosenberg *et al.* 2002, Fig. S1a; Jakobsson *et al.* 2008, Fig. 1a; Rosenberg 2011, Fig. 4a and Table 2; The 1000 Genomes Project Consortium 2015, Fig. 1a). Finally, Fig. 5 illustrates that pattern probabilities can be considered locally as a function of genomic position; this form of analysis can also suggest signatures of population-genetic processes such as balancing selection in the HLA region (Fig. 6).

Our analysis has made use of dense human genomic data. For genomes with a higher density of variants than the human genome, shorter window sizes may be convenient for measurement of pattern probabilities. For lower-density data, longer window sizes might be required for accumulating enough variable sites to accurately measure pattern probabilities. Even in the data we have examined, data quality might vary across windows; this problem might affect the HLA region, in which high variation levels can lead through technical artifacts to biased estimation of allele frequencies (Brandt *et al.* 2015). The window size can be tuned appropriately to the analysis of interest.

Our observation that allelic types are generally rare in some human groups and unobserved in others, or common in most or all groups—here seen with a rarefaction method—has been consistently observed across datasets and choices of population groups (Cavalli-Sforza *et al.* 1994; The International HapMap 3 Consortium 2010; Rosenberg 2011; The 1000 Genomes Project Consortium 2015; Biddanda *et al.* 2020). Analyses enabled by a focus on pattern probabilities, with the improvements from the sample-size correction introduced here, provide new approaches to emphasizing and visualizing this fundamental result in human evolutionary genetics.

## Supplementary Material

iyad070_Supplementary_Data

## Data Availability

We downloaded publicly available data from the 1000 Genomes FTP site: http://ftp.1000genomes.ebi.ac.uk/vol1/ftp/data_collections/1000G_2504_high_coverage/working/20190425_NYGC_GATK/. All code used for the analyses is available on GitHub: github.com/djcotter/rarefaction-rare-vs-common. Supplemental material is available at GENETICS online.
