# Supplementary material for: A rarefaction approach for measuring population differences in rare and common variation
Source: Genetics. 2023 Apr 19;224(2):iyad070. doi: 10.1093/genetics/iyad070 (PMC10213490; doi:10.1093/genetics/iyad070)
Supplement: iyad070_Supplementary_Data [file iyad070_supplementary_data.zip › Figure_S1_GENETICS-2023-306051.pdf]

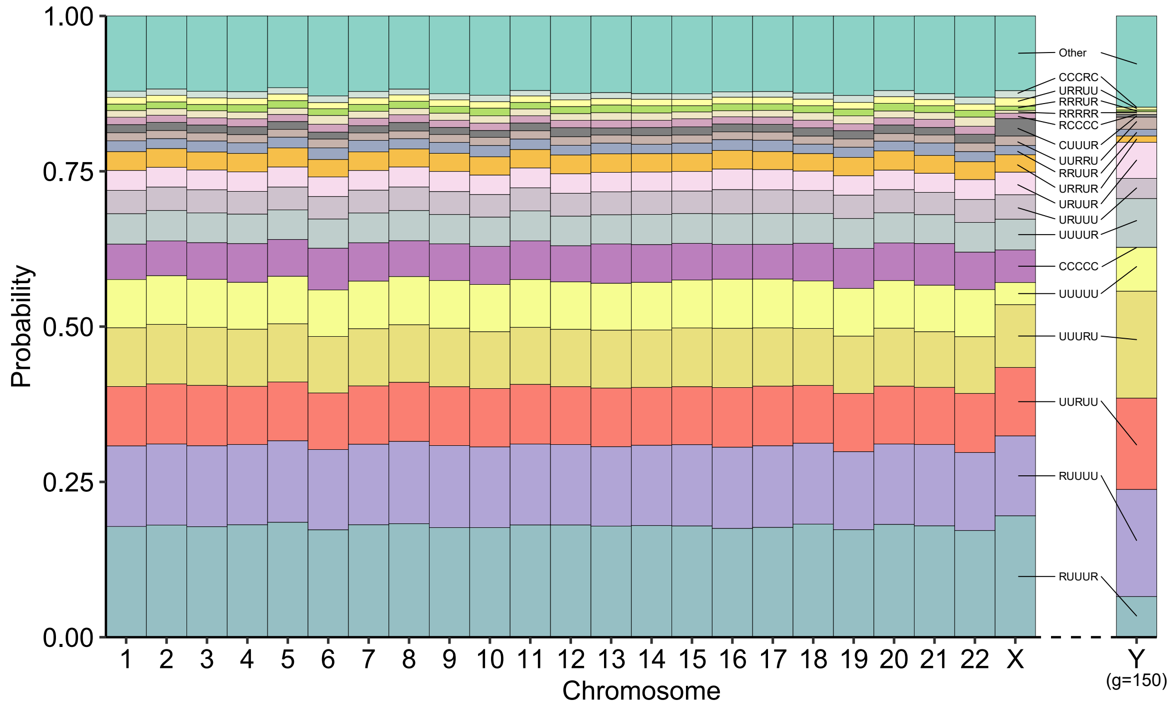

Figure S1: Probability that the globally minor allele at a locus has a given geographic distribution pattern, considering each of 22 autosomes and the two sex chromosomes. Probabilities are calculated using all non-singleton SNPs on each chromosome and $g = 500$ ($g = 150$ for the Y chromosome). Patterns that are present at greater than 1% frequency on any autosome are indicated, with all other patterns grouped into the "Other" category.
