# Supplementary material for: A rarefaction approach for measuring population differences in rare and common variation
Source: Genetics. 2023 Apr 19;224(2):iyad070. doi: 10.1093/genetics/iyad070 (PMC10213490; doi:10.1093/genetics/iyad070)
Supplement: iyad070_Supplementary_Data [file iyad070_supplementary_data.zip › Figure_S2_GENETICS-2023-306051.pdf]

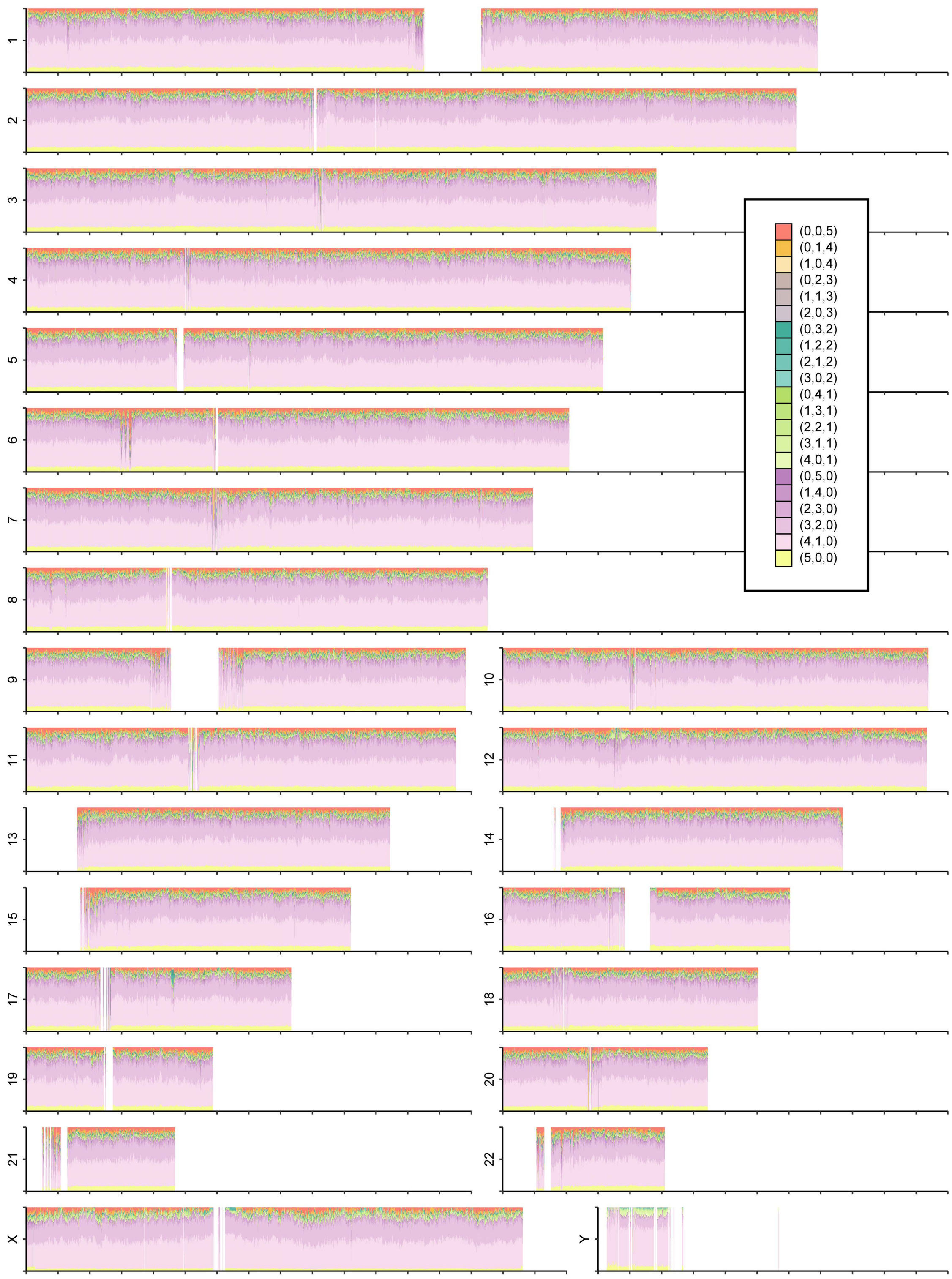

Figure S2: Probabilities for groups of patterns for minor alleles across all 22 autosomes and the two sex chromosomes, in samples containing $g = 500$ alleles from each super-population, averaged across all non-singleton loci in non-overlapping 100-kb sliding windows ($g = 150$ for the Y chromosome). Ordered triples are written $(|U|, |R|, |C|)$, with the entries representing the numbers of super-populations in which allelic types are unobserved, rare, and common, respectively. Triples are grouped by color, varying within classes with a given number of super-populations in which allelic types are common. Each X-axis tick mark corresponds to a distance of 10 Mb.
